# Between Ambitious Strategies and Reality: The African Union Strategy on COVID-19 Vaccine

**DOI:** 10.3390/epidemiologia2040042

**Published:** 2021-12-14

**Authors:** Amr ElAteek, Shimaa A. Heikal, Ludmila Rozanova, Antoine Flahault

**Affiliations:** 1 Global Health Institute, The University of Geneva, 1211 Geneva, Switzerland; Amr-Abdelhady.Elateek@etu.unige.ch (A.E.); ludmila.rozanova@unige.ch (L.R.); antoine.flahault@unige.ch (A.F.); 2 Public Policy and Administration Department, The School of Global Affairs and Public Policy, The American University in Cairo, New Cairo 11835, Egypt

**Keywords:** COVID-19, vaccines, vaccination strategy, African Union, COVAX

## Abstract

A key learning lesson from country responses to COVID-19 pandemic was the impact of the strategies that are followed on combating the pandemic. Since the development of different vaccines and their supply globally, most countries have developed their own strategies to effectively provide sufficient doses for their population and start the vaccination process with the best outcomes. In this perspective, we review the African Union vaccination strategy by exploring the implementation of the strategy and its outcomes. We report on the strategy initiatives, vaccines distribution, administration, and the impact on new COVID-19 cases in African countries.

## 1. Introduction

For the past few years, speculations have been raised on how the world is prepared for coping with the next pandemic that would be hitting the world or, as been called by then, “disease x” [1]. All indicators showed that a global health crisis is approaching since a noticed increase in the number of emergent and recurrent epidemic hotspots [2] and the impact of climate change that became more tangible recently [3]. Unfortunately, the African continent had the lion’s share of those health crisis events, like Cholera, Zika, and most memorably, the Ebola outbreak that has been affecting the west African countries the most [4].

The epidemic that erupted in 2014 caused 28,616 infections and 11,310 deaths reported from Guinea, Liberia, and Sierra Leone, in addition to 36 cases and 15 deaths from other countries [5]. Accusations have been pointed against policymakers and stakeholders in the delay for taking bold actions toward controlling the situation by then [6]. Yet the joint external evaluation that was conducted to assess the situation followed by the case management interventions, infection prevention control practices, surveillance and contact tracing, good laboratory service, and dignified burials and social mobilization that were done by the health authorities in the affected areas in coordination with the world health organization (WHO) represented a great model on health emergency response and helped effectively in the management of the epidemic [7]. Although the efforts that have been done to compress the clinical trials for the development of the Ebola Vaccine faced so many obstacles like; the deperiortization of the event compared to other public health issues, hence the lack of pharmaceutical companies interests and consequently the lack of funding which led to the slowdown in the progress of developing an effective vaccine [8], yet it gave a great lesson on how to act appropriately in the future similar events and to create a fast track that would speed up the process of registration and making available required medicines and vaccines during acute public health emergencies of international concern [9].

The year 2020 came to make all the previous predictions come true, with the COVID-19 pandemic erupted in China and spread rapidly to the rest of the world [10]. As of mid-March 2021, after nearly a year of World health organization (WHO) declared COVID-19 as a pandemic, more than 120 million cases of coronavirus infections have been recorded across the world and still counting [11] The actual numbers may be more than the declared numbers due to unrecorded cases or the lack of adequate monitoring and tracking mechanisms [12,13].

Most countries began to take the necessary countermeasures to treat the infected cases and control the spread of the pandemic. The interventions varied from closing the borders, applying full or partial lockdown, promoting preventive social practices, and implementing required measures that would mitigate devastating socio-economic situations [14]. Despite the measures that have been taken, the lack of a unified treatment protocol led to the emergence of some medical malpractices, like off-label drug use [15]. Hope has been rising since the announcement of The United States Food and Drug Administration (FDA), and European Medicines Agency (EMA) approved COVID-19 vaccines using the Emergency Use Authorization [16]. Countries started to put forward their plans to ramp up all their efforts to secure resources to procure and distribute the required doses to inoculate their population against COVID-19. The competition, either through global/regional alliances and cooperation or through direct bilateral agreements with the manufacturers, made a scene of an ardent race. This race aims at taking a significant step in the fight against the pandemic. Yet, due to the difference in capabilities and resources of each country, inequity in distribution and access to vaccines has been witnessed, despite all the efforts exerted through the COVAX initiative to ensure fair access to low-income countries.

The first COVID-19 case detected in Africa was laboratory-confirmed and reported on the 14th of February 2020 for a German tourist who came to visit Egypt during the vacation [17]. Despite the slow to spread in Africa compared to other continents [18], the impact of COVID-19 has affected most African countries gradually, which urged the African Union (AU) to advance the efforts to deploy the proper vaccination strategy, identifying the continent’s limited infrastructure and resources.

This article explores the AU vaccination strategy and provides an overview of the progress of the strategy implementation in the AU countries through reviewing the process that led to the development of the strategy, its implementation, and results obtained. The purpose of this study is to (1) provide insights into the coherence of the strategies adopted by the AU, (2) how they were implemented to tackle the pandemic, and (3) how successful was the strategy and adopted policies during the rollout of the vaccine, scale-up as well as, (4) their impact on the decreasing the new COVID-19 cases and deaths.

## 2. Background and Case Presentation

### 2.1. Africa Demographics

Covering nearly one-fifth of the earth’s total land surface with approximately 30,365,000 km^2^, the African continent represents the second largest continent in the world after Asia [19]. According to the last estimate in 2021, Africa counts for being the home of more than 1.340 billion people [20] and has the title of being the youngest continent as 60% of its population is below the age of 25 [21].

The official languages of the AU are English, French, Arabic, and Portuguese. Yet, a massive linguistic diversity has been found with an estimate of 1500 languages [22], the thing that reflects on the variations in the cultural and religious norms of the continent.

### 2.2. Political Overview

For a long period, Africa has been suffering from colonization, wars, and political instabilities. Despite the end of the imperialistic era in Africa by 1975, the transition stage of shifting from colonial governments to independent states has been characterized by political disturbances, conflicts, violent events, civil wars, and geographical separations [23]. The political hardship that most of the countries in Africa have witnessed represent the main obstacle to the continent’s economic development [24].

### 2.3. Economic Impact

The African economy can be categorized into three main categories: the first one is the oil-producing countries [25]. The economic regression in this sector has been witnessed due to the decrease in worldwide mobility, which has led to the decline in crude prices and has consequently affected the economic situation of those countries. Likewise, the tourism-dependent countries have been extensively affected by the limitations that have been imposed on the travel regulations. On the other hand, on reviewing the last category, they would be best described as the diversified economies. Despite the deterioration that has been witnessed in this type of economy, the diversification in their sources of structuring the economy has improved their ability to cope with the pandemic impacts effectively [25].

However, If COVID-19 continues to spread, the World Bank estimates that economic growth in sub-Saharan Africa will decline from 2.4% in 2019 to between −2.1 and −5.1% in 2020, causing the first recession in the region in 25 years [26]. This forecast was evidenced by an actual contraction in 2020 based on the data that arose from the sub-Saharan African region [27]. On the other hand, the economic growth in the continent is forecasted to be regained between 2.3 and 3.4% in 2021 based on the interventions and actions adopted by the countries [28]. Vaccines can interrupt this disruption of national economies in Africa and prevent further reversal of the gains made over the past several decades.

### 2.4. Health Systems

Before the COVID-19 pandemic, African health systems were already enduring the impacts of the challenges that are obstructing the process of healthcare infrastructure improvements as underfunded health systems, the inequitable access to adequate healthcare services, the lack of the required capacities, and the socio-economic situations that cause a rise in vulnerable groups, which remained unidentified or unincluded in the health systems.

A study conducted recently by The Center for Disease Dynamics, Economics and Policy estimated the critical care capacities in 54 African countries. One of the study’s main findings was the average of the intensive care unit beds, which was found to be 3.10 beds and 0.97 ventilators per 100,000 people [29].

This pre-COVID-19 situation, though, does not deny the massive efforts put in by many African countries to improve their healthcare systems through the development of national strategies aligned with the United Nations 2030 sustainable development health goals, aiming at improving health outcomes through promoting health security, implementation of universal health coverage by increasing access to adequate healthcare services, essential medicines, and vaccines for all, without leaving anyone behind [30].

The continent’s capability to contain previous outbreaks and the spread of non-communicable diseases cannot be cited unless describing the health systems’ fragility. On the other hand, learning from past lessons is considered to be a good trait that characterizes the continent. So far, the FDA has approved the emergency use of 3 vaccines [31], with 97 other vaccines at the clinical development stage [32]. All these efforts make it possible to favor winning the battle against the pandemic. Yet, challenges like intellectual property and sharing the know-how of vaccine manufacturing remain a stumbling block that prevents the mass production of vaccines to the required extent for fulfilling the current world need [33].

## 3. Materials and Methods

### 3.1. Study Design

This descriptive study is conducted based on real-time information reported by various publicly available data sources. However, given the dynamic and rapidly evolving situation, and the drastic change in the vaccination statistics between African countries, the study is limited to the specific time between February 2020 and June 2021 to focus on the early response and initial vaccination strategies of the African continent during this early timeframe.

### 3.2. Literature Search and Data Collection

We performed a rapid literature search and identified peer-reviewed papers and public reports that studied and reported the AU vaccination strategies and the national and subnational responses of the African countries in implementing the strategies and scaling-up procedures. Data about the vaccine rollout, vaccinated population, new COVID-19 cases, and challenges faced during the implementation of the vaccination strategies were extracted from various publicly available data sources, such as the official websites of the AU, Africa Centers for Disease Control and Prevention (ACDC), AU press release, COVAX initiative through Gavi the vaccine alliance, official national African countries websites, higher educational institutions, and the news agencies.

### 3.3. Reviewing Process

To achieve the main objective of assessing the impact of the strategy, we reviewed different components of the vaccination strategies at the African regional level as well as the national levels, including:

1. The coordination plans between AU, countries, and various facilities to provide sufficient vaccines for the African population;
2. The planning of different phases of vaccination, including rollout and scale-up;
3. The prioritization of populations in the rollout phase;
4. The vaccines approved and the logistics of vaccines distribution;

The impact of the strategy was then assessed through measuring the following:

1. The vaccination provider enrollment;
2. Doses distributed;
3. 1st and 2nd doses administered;
4. Vaccination coverage;
5. The decrease in COVID-19 cases in AU countries after vaccination.

### 3.4. Figures

**Figure 1:**
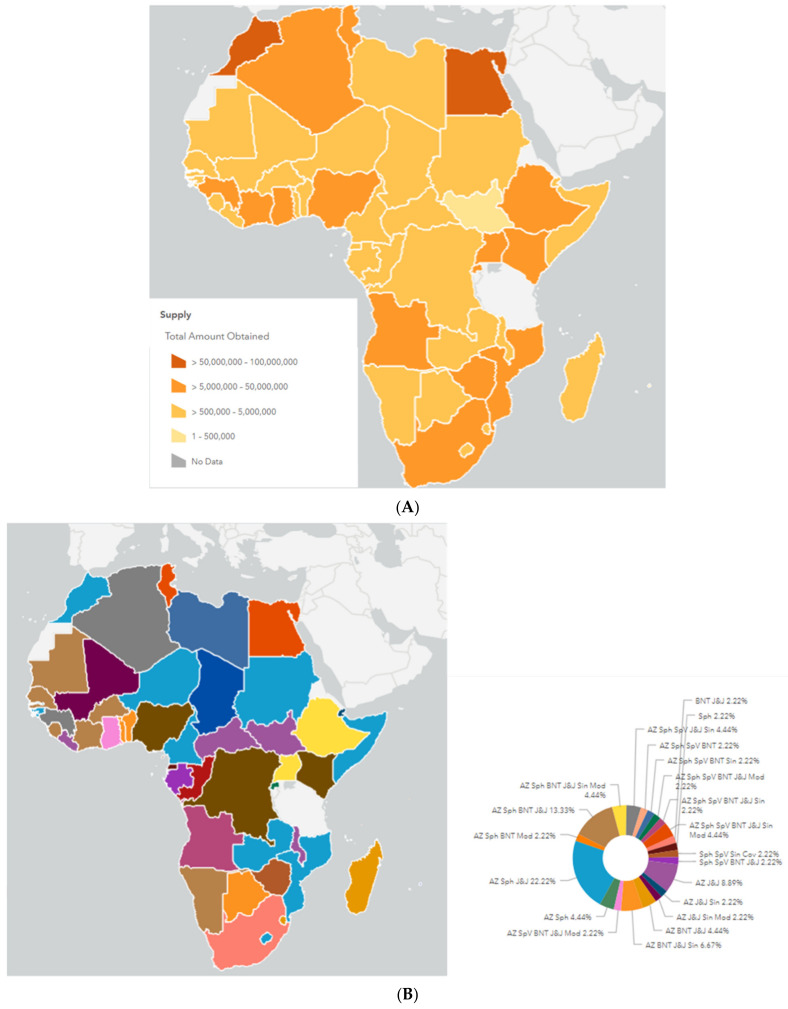

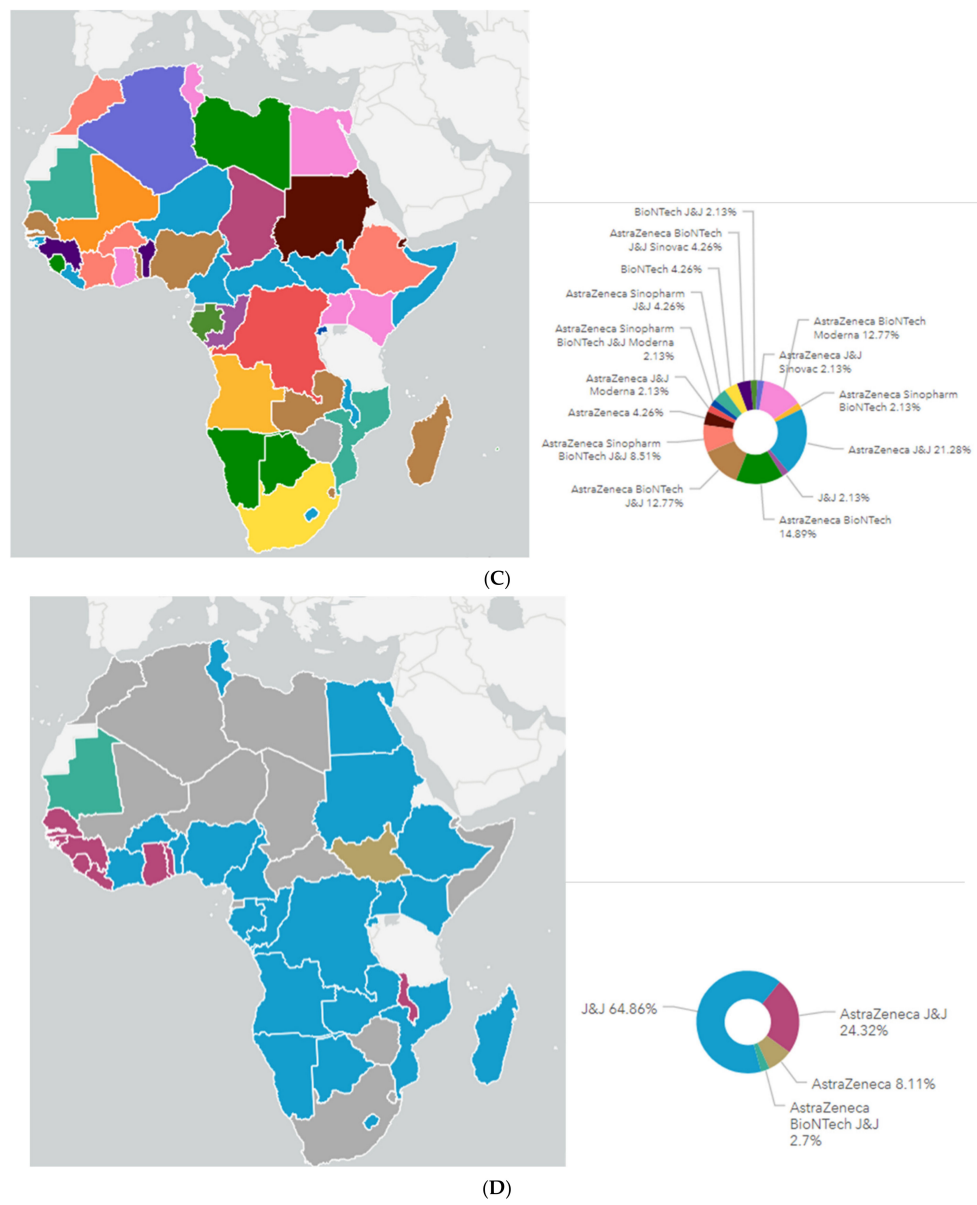

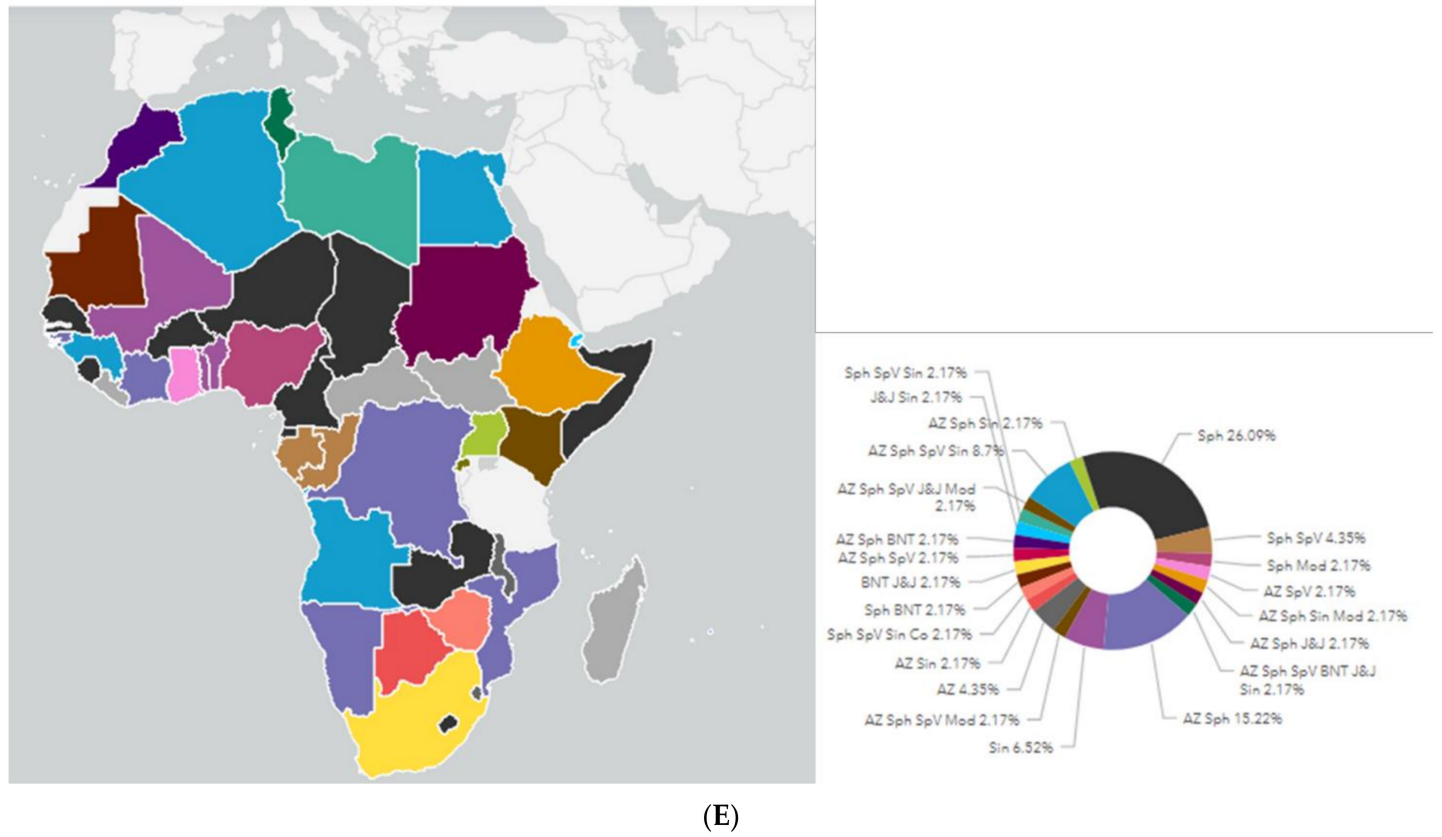
COVID-19 vaccine distribution in Africa until 30 June 2021. (**A**) A map showing the number of doses applied to each country. (**B**) A map showing the types of vaccines distributed to each country. (**C**) A map showing the countries received COVAX vaccines. (**D**) A map showing the countries received AVATT vaccines. (**E**) A map showing the countries received vaccines bilaterally; (AZ: AstraZeneca, Sph: Sinopharm, SpV: Sputnik V, BNT: BioNTech, Sin: Sinovac, Mod: Moderna, Cov: Covaxin). Source: Africa CDC vaccine board [34].

**Figure 2:**
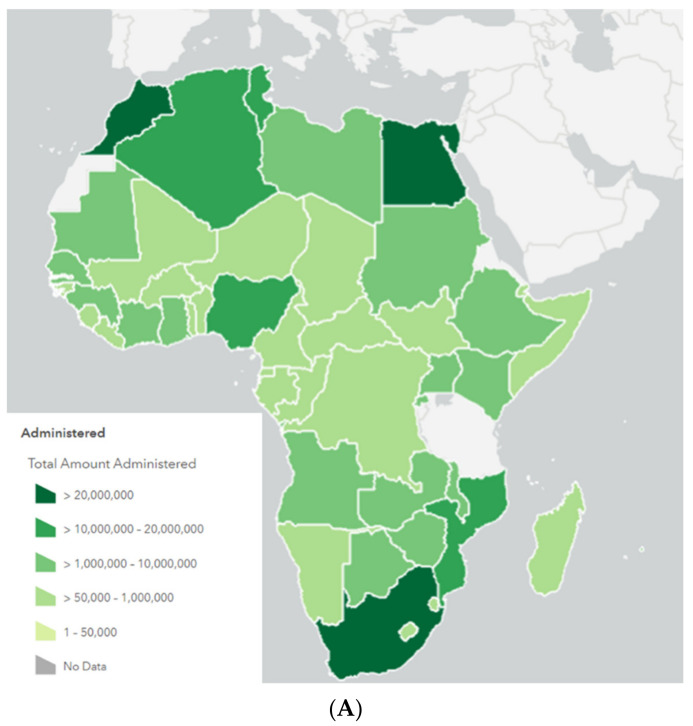

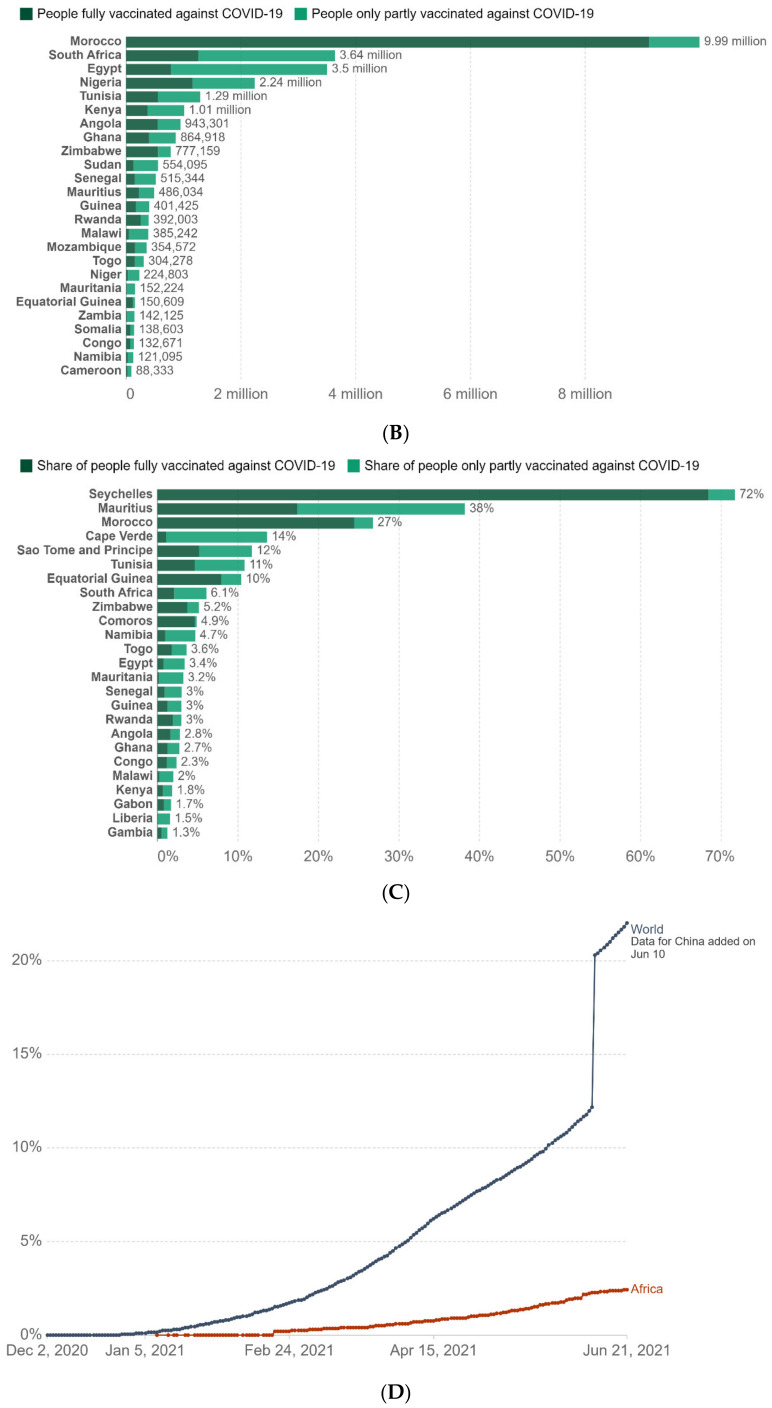
COVID-19 vaccine administration in Africa. (**A**) A map showing the number of doses administered in each country. Source: Africa CDC vaccine board [34] (**B**) The number of people who received one or two doses of vaccine in each African country until 30 June 2021 (the highest 25 countries are represented). (**C**) Share of the total population in each African country who received one or two doses of the vaccine until June 2021 (the highest 25 countries are represented). (**D**) A comparison between the vaccine coverage in Africa and the whole world until June 2021, source of (**B**–**D**): Coronavirus vaccination statistics and research [35].

**Figure 3:**
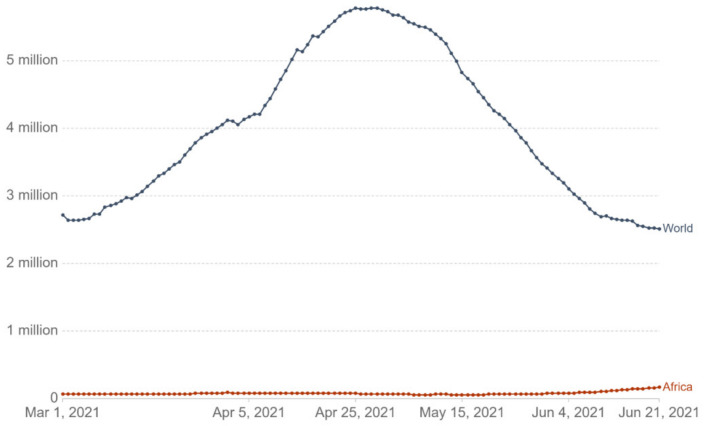
New weekly confirmed COVID-19 cases in Africa compared to the world after starting the vaccination campaigns (March 2021 to June 2021), each data point is the sum of COVID-19 cases reported within a week, source: Coronavirus vaccination statistics and research [35].

We generated Figure 1, Figure 2 and Figure 3 to demonstrate the vaccination distribution and coverage through all the continent in order to be able to visualize the details of the early responses. The figures were generated through the data published in Africa CDC vaccination dashboard as well as Oxford’s project -our world in data-. We applied the appropriate filters needed to get the required graphs representing the African continent [34,35].

## 4. Results

### 4.1. Non-Pharmaceutical Interventions

As of the 29th of June, African countries reported 5,442,372 COVID-19 cases and 141,698 deaths [36].

Since the start of the pandemic, African countries have had many regulatory interventions that were implemented, including lockdowns and curfews. The extent of implementation of these regulatory actions has differed from one country to another, based on the pandemic situation in the country, including full and partial lockdowns, prohibition of gatherings, and closure of unessential shops, restaurants, and cafes. The duration of implementing these regulatory actions also differed in each country, starting March 2020 and up until September 2020. However, it is not that those interventions effectively helped control virus spread [37]. Those interventions were reinforced by supportive packages offered to the African citizens to mitigate the drastic socio-economic impact caused by the pandemic [37].

### 4.2. AU Response to the Pandemic

Through various high-level meetings and initiatives, the AU, and its specialized technical arm for public health (ACDC), has been working closely with the African member states to support their response and to expand the continent’s resilience capabilities to cope with the spread of the pandemic.

A joint continental strategy for COVID-19 response was developed to prevent severe illness and death from COVID-19 infection in the African Member States and minimize the social disruption and economic consequences of COVID-19 outbreaks. The joint strategy worked on Coordinating the efforts of Member States, AU agencies, World Health Organization, and other partners to ensure synergy and minimize duplication. Besides promoting evidence-based public health practice for surveillance, prevention, diagnosis, treatment, and control of COVID-19. The joint strategy was adopted by the African ministers of health on 22 February 2020, during the first emergency ministerial meeting on COVID-19 [38].

In February 2020, the African task force on coronavirus (AFTCOR) was launched, representing the cornerstone in collaborating the efforts for COVID-19 preparedness and response among the African countries. By identifying the main six technical areas of focus, working groups were formed on to include surveillance and screening at points-of-entry, laboratory diagnosis, Infection prevention and control measures in healthcare facilities, Clinical management of severe COVID-19 infection, risk communications, and Supply chain medical supplies [38].

To support the joint continental strategy through increasing the capacity for testing COVID-19 to ensure the implementation of evidence-based policy-making process based on the interchangeable situation, Africa CDC has launched the Partnership in May 2020 to accelerate COVID-19 Testing in Africa (PACT). By mobilizing required experts, community workers, and resources to Test, Trace, and Treat the COVID-19 cases, the ACDC set its sight on accomplishing the conduction of 10 million tests for COVID-19. In addition to the training of 100,000 healthcare workers and the deployment of 1 million community health workers to mitigate the impacts of the pandemic in an ongoing project. The initiative also aspired to develop a continent-wide procurement platform for laboratory and medical supplies, which have been achieved and launched in July 2020 [39].

The harsh conditions caused by the pandemic and the excessive need for medical equipment and protective tools like facial masks and personal protective equipment, alongside the diagnostic kits, created a necessity for creating a platform that would fulfill the needs of the continent during emergencies and during regular times as well. The online Africa Medical Supplies Platform (AMSP) came to close the gap by creating a partnership with the African Export-Import Bank (Afreximbank) and United Nations Economic Commission for Africa (ECA) with the support other partners across the globe [40].

Through its initiative, the AMSP launched the COVID-19 vaccine pre-ordering program for the 55 African countries. The program aims to achieve herd immunity by securing the vaccination for 60% of the African citizens. Through negotiating with vaccine manufacturers, the program can secure the required doses of vaccines with competitive prices that would be suitable for the continent’s economic situation. This initiative will ensure equitable access for African member states to procure the required doses of vaccines for their citizens. It is important to mention the support that the Aferximbank has provided in facilitating the payments by offering advance procurement commitment guarantees that would give support of up to US$2 billion to the manufacturers on behalf of the Member States [41].

As a step toward closing the gap of equitable access to vaccines and medical supplies, not only for the current pandemic but also for any future health threat, the AU conducted a set of high-level meetings inviting presidents, policymakers and technical experts in the context of expanding the continent’s vaccine manufacturing capabilities [42]. The meetings yielded the launch of Partnership for African Vaccine Manufacturing (PAVM), which included the signing of two agreements with the Coalition for Epidemic Preparedness Innovations (CEPI) and Afreximbank. The two agreements aim for increasing the increase in vaccine development research and boosting the local manufacturing capacities in the continent [43].

### 4.3. Medical Supplies Donations

One of the main gaps that current pandemic exposed was the solidarity among the nations and the support by countries and entities to cope with negative socio-economic impacts. The AU was able during the first and second wave of the pandemic to coordinate all the efforts to allocate donations of medical supplies and medications to the African member states to support their countermeasures facing the pandemic. Donations from governments like China and Canada, in addition to foundations like Jack Ma foundation [44], contributed effectively to achieving the goal set by the Joint continental strategy of preventing severe illness and death from COVID-19 infections. As of mid-April 2020, The AU received the third wave of donations that included 4.6 million masks, 500,000 swabs, and test kits, 300 ventilators, 200,000 sets of protective clothing, 200,000 face shields, 2000 temperature guns, 100 body temperature scanners, and 500,000 pairs of gloves. Those tools and kits were distributed to the African countries based on their needs [45].

### 4.4. COVID-19 Vaccine Strategy Development

The heterogeneous increase of cases in the African countries was a predictor of the uncertainty of the continent’s future, particularly after the massive increase in the reported deaths by December 2020. Hence, a global solution was the optimal decision as the pandemic was a global health emergency, and no country will be safe until all the countries are protected [46]. Accordingly, a vaccination plan for the African countries was necessary to combat and contribute to ending the pandemic and to overcome the humanitarian and economic crises that have developed through the extended lockdown.

Realizing the mess that has been caused by the COVID-19 pandemic across the globe, the AU took a step toward unifying the vision of the African member states through drawing the bold framework via the COVID-19 vaccine strategy.

The strategy, which was launched after a virtual conference for the African ministers of health that was conducted on the 24th and 25th of June 2020 [47], came to focus on the means of securing the required doses of vaccines by coordinating the efforts with global partners. The strategy also highlights the challenges and obstacles that would be faced during the procurement process and the following rolling-out stage.

#### 4.4.1. Initial AU Vaccination Efforts

Following the rapid expansion in the COVID-19 situation, Africa CDC initiated some efforts to mitigate the impact of the pandemic. A comprehensive region-wide strategy was the primary step to help all the African countries in preventing the transmission of the virus among their populations, preventing the death of COVID-19 cases through providing improved healthcare procedures, and avoiding the social and economic implications of the epidemic [48]. The rollout of an efficacious vaccine that can be safely and widely accessible by all African countries was the key to implementing the strategy’s three objectives.

##### Developing the First Vaccination Strategy

In June 2020, a conference was hosted by the Africa CDC to discuss the vaccine needs in the region, as well as the opportunities for developing, manufacturing, distributing, and taking up the vaccine [49]. More than 3000 leaders and technical experts agreed on the urgent need for immunizing the African population, and based on their recommendations, the first strategy for vaccination of the African continent was developed with a goal of immunizing at least 60% of the African population. The strategy had some objectives, including accelerating the involvement of Africa in the vaccines’ clinical development, guaranteeing the accessibility of African countries to the globally available vaccines, and overcoming any barriers that might hinder the widespread distribution of vaccines in Africa [48]. To achieve these objectives, The AU and Africa CDC played an essential role in the implementation roadmap to coordinate between African and global organizations such as member states, regulatory agencies, and other partners.

##### Fair Allocation of Vaccines

Various technical and ethical concerns arise during the implementation of the vaccination strategy, particularly in making the prioritization decisions. The scarcity of resources and vaccines and the limited time were elements to be considered, along with the assessment of target groups before setting up vaccines [50]. Accordingly, in December 2020, Africa CDC, and the South African Medical Research Council (SAMRC) developed a framework as a guideline for the fair allocation of vaccines. The framework drew on the expertise and input of more than 1300 policymakers, community advocates, public health experts, and ethicists who worked together to ensure the fairness and equity of vaccine distribution. Although allocating such scarce resources like the COVID-19 vaccines required considerations of the maximized benefits and giving priority to the most vulnerable populations, the equitable distribution of vaccines among Africa was also crucial to develop herd immunity and protect the whole population [50]. Hence, the vaccination program aimed to decrease morbidity and mortality of the cases with several values during the allocation of vaccines, including (1) Affirming the others’ humanity through considering societal benefit and the common good when allocating the vaccines; (2) Depending on the best evidence that ensures the survival of the community like when prioritizing the vaccination of healthcare workers; (3) Considering social solidarity issues and the bonds between communities to avoid creating new inequalities; (4) Allowing the active community engagement in the allocation decisions to promote trust [50].

##### The African Vaccine Acquisition Task Team

The African Vaccine Acquisition Task Team (AVATT) was established in August 2020 to lead the continent’s efforts to access vaccines and achieve herd immunity. The WHO supported the team and worked together with the COVAX facility to secure the required vaccine doses for African countries. By Jan 2021, and to achieve the goal of immunizing 60% of the population, AVATT acquired over 270 million doses of COVID-19 vaccines from Pfizer, Johnson & Johnson, and AstraZeneca providing equitable access to doses for the 55 member states of the AU [51]. In mid-March 2021, AVATT received a batch of the Oxford-AstraZeneca vaccine as a donation and shipped around 925,000 doses to 13 AU member states. Despite the limited expiration of the vaccine, Africa CDC decided that countries should vaccinate their populations quickly as there is an urgency to save people’s lives. In addition, the Africa CDC was able to redistribute the vaccine consignments of the countries that could not use them to other member states [52,53]. AVATT also had a historic agreement with Johnson & Johnson to provide access for 220 million doses of their single-shot vaccine to all the continent’s member states with a potential to order more than 180 million doses [54]. All the vaccines will be manufactured in South Africa’s giant pharmaceutical plants to be supplied to the countries within 18 months in a future perspective. Moderna, BioNtech and Pfizer have started the process of initiating facilities to produce mRNA vaccines in the African continent by 2022. Moreover, the production capacities of the on-work facilities are estimated as 50 million doses per year and they will sustain producing other types of vaccines for the African continent, such as Malaria vaccines [55].

#### 4.4.2. Cooperation with COVAX Facility

Given that international collaboration is essential for the fair distribution of COVID-19 vaccines, and to ensure that the financing of vaccines will be then distributed in transparent and coordinated manner, the WHO led different arrangements with organizations and stakeholders to ensure the safe and efficient allocation of vaccines [56,57]. One of these initiatives is the COVAX facility, which was delineated in September 2020 as a multilateral initiative led by Gavi Vaccine Alliance (Gavi), the World Health Organization (WHO), and the Coalition for Epidemic Preparedness Innovations (CEPI) [58]. COVAX was a voluntary arrangement that aspired fair and equitable access of vaccines to all countries by helping them invest in the vaccines while developing the required political and logistical infrastructure for the distribution of the vaccine [59].

In addition, the COVAX facility has made investments in several vaccines that were shown to be promising. The pooling of purchasing power from all the participating countries will accelerate the access to vaccines once developed. Moreover, to overcome the obstacle of the initial limited supply of vaccines, the WHO advised that all countries should receive doses once an effective and safe vaccine is approved. Receiving doses proportional to the country’s population size will allow countries to prioritize their vulnerable populations and start immunizing them. The framework of the fair allocation of vaccines through COVAX had the goals of initially allocating proportional doses of vaccines that cover 20% of the country’s population, and to expand the coverage afterwards in the follow-up phase to cover all the populations [60].

##### The Access to COVID-19 Tools Accelerator

The Access to COVID-19 tools (ACT)-Accelerator was a partnership launched in April 2020 by the WHO and partners including UNICEF and Gavi to rapidly e tests, treatment, and vaccines and end the acute phase of the pandemic [61]. The ACT-Accelerator had four main pillars that are vital for fighting the pandemic, including diagnostics, treatment, vaccines, and health system strengthening. The access and allocation mechanisms to these tools are also fundamental to the goals of ACT-Accelerator. Immediately after the launch of ACT-Accelerator, UNICEF became a partner in the COVAX initiative to achieve the vaccines pillar through COVAX facility [62,63]. The goal was that COVAX scales up the delivery of vaccines to at least 2 billion doses by 2021 [64].

##### COVID-19 Vaccines Advance Market Commitment (COVAX AMC)

In June 2020, Gavi launched the COVAX AMC during the global vaccine summit as a building block of the COVAX facility. COVAX AMC was aimed to be the financing tool to support the 92 low- and middle-income countries participating in the COVAX facility [65]. With the coordination of country readiness, AMC will assure the protection of the most vulnerable populations in a country regardless of their income level. Accessing COVID-19 vaccines through COVAX AMC required countries to fulfill several steps starting with the confirmation of participation in the COVAX facility via signing a vaccine request. Then comes the step of preparing and introducing countries to the vaccines which are led by the WHO, UNICEF, and Gavi guidelines. After that, the readiness of the county to receive and use the vaccines is assessed to establish a suitable framework for disseminating the vaccines. The allocation of vaccines will consider the supply and demand of each country to ensure that the distribution follows the fair allocation goal of COVAX AMC strategy. Finally, participant countries provide the required documents to COVAX to release the assigned doses and start the vaccine delivery [66].

### 4.5. Vaccine Distribution in Africa

In February 2021, the COVAX AMC started their campaigns to deliver COVID-19 vaccines to Africa starting with Ghana and Cote ‘d’Ivoire with 60,000 and 504,000 doses, respectively. All the distributed doses were from the Oxford-AstraZeneca vaccine, while the Pfizer vaccine was supposed to be delivered in later distribution batches [67,68,69].

By the end of April 2021, COVAX has supplied many African countries with the initial vaccination doses, mainly the AstraZeneca vaccine (over 92% of all the supplies) [34]. So far, 44 African countries are now participants of the COVAX facility and are receiving the vaccine doses from different vaccines that have been approved for emergency use, including AstraZeneca/Oxford, Johnson & Johnson, Moderna, Pfizer/BioNTech, Sinopharm, and Sinovac [70]. In addition, 37 countries have received vaccines bilaterally, and 13 countries have received AVATT vaccines (Figure 1).

However, despite all these efforts, as of mid-April 2021, below 2% only of the African population [71] was vaccinated, indicating the continent’s struggle to achieve the 60% vaccination goal [34]. This lagged situation in the vaccination rate could be referred to for various reasons, most notably the Indian move toward halting vaccine exporting as a result, leading to a delay in COVAX global supply that are significantly assigned for African countries [72]. Additionally, the African local vaccine manufacturing capacities are at a very low pace for fulfillment of the continental needs [73].

### 4.6. Vaccination Coverage

As of the beginning of July 2021, while most countries were progressing toward inoculating a major sector of their population, the African continent was struggling to reach nearly 2% of its population, with 13 million out of 31.6 million doses delivered so far having been administered [74]. Additionally, the low vaccination rate was accompanied by an asymmetric pattern in distribution since 93% of the doses were given in 10 countries of the continent [74]. The high rates of vaccination rollout in those countries were due to the implementation of strategic planning for identifying priority groups and the effective utilization of resources to quickly achieve the needed outcome. However, it is important to consider other factors that would participate in the false perception of the overall rate of vaccination, like the country’s population compared to vaccination coverage. For instance, Seychelles has the highest vaccination rate, with 140 doses administered per 100 individuals. Yet, it is important to note the Seychelles population is nearly reaching 100 thousand inhabitants [75]. On the other hand, Morocco has a rate of 47 doses per 100 persons, considering the total population is nearly 37 million, this gives a good indication of the progression of the ongoing vaccination coverage (Figure 2) [75].

### 4.7. COVID-19 Cases in Africa after Vaccination

With the vaccine rolling out across the globe, and the notable decrease in the cases for vaccinated populations. This was documented in many countries, especially in the United States, which has experienced, by June 2021, a significant decline in cases that reaches 94% compared to the pandemic peak in January [76]. With the expansion in the vaccination coverage, data have shown how effective vaccines are, especially for elderly people. According to the Center of Disease Control (CDC), as of May 2021, 69% of American adults over the age of 65 were fully vaccinated against COVID-19. Consequently, a large decrease in infections, hospitalizations, and deaths was seen [77]. On the other hand, Africa was not witnessing the same level of case decline, on the contrary, as of Mid-June 2021, COVID-19 cases jumped 44% to 95,000, while fatalities rose 20% to 1400 over the previous week across Africa (Figure 3) [78].

## 5. Discussion

The uneven vaccination rate among many countries across the globe may be the main cause of the gap between developed and developing countries, particularly in Africa. Indeed, the lack of access to vaccine and the weak supply chain infrastructure represent one of the main challenges that resulted in the current situation of low vaccination coverage. Yet there are other factors, persistent and emergent if we were to categorize them. One of the persistent challenges we refer to is the low socio-economic situation the African continent has been suffering from throughout history. The impacts of this status are much more tangible whenever an emerging problem—either health-related or non-health-related—occurs. Additionally, the pandemic revealed a lot about some of the obstacles the health systems in Africa are suffering from. The weak health infrastructure and the lack of accurate data may represent the main causes of the dereliction in managing cases properly and hence explains the high mortality rate seen in the COVID-19 cases on the African continent. On the other hand, the emergence of new variants in late 2020, especially the South African and Indian variants (Beta and Delta variants as labeled by the WHO), has created a great challenge that has the highest impact in Africa [79]. The new variants were mutants of the wild type and were shown to be more transmissible than the original variants, which made their response to the developed vaccines questionable [80]. Although there is still no evidence to suggest that the new variants are more lethal, the data gathered confirmed that the new variants differ in their intensity and rate of transmission.

Overall, the AU vaccination strategy seems to be very optimistic, with major challenges that might hinder the achievement of the main goal of vaccinating 60% of the population, even by 2023. Major health system reforms, as well as better resource allocation, will be crucial for countries to achieve better results. On the other hand, while the continent should seek more international collaborations, the international community shall be more effectively responding in order to guarantee the supply of sufficient doses to the African countries with suitable costs.

## 6. Conclusions

Despite the fragility and vulnerability of the health systems that Africa has suffered from for decades, the COVID-19 pandemic came with expectations that were in favor of the failure to contain the pandemic and turn the region into a hotspot for the virus and its variants; the response of the African countries, supported by ACDC, showed a great model of resilience, collaboration and solidarity that affected the pandemic situation compared to other regions of the world. Through the initiatives that the AU has created, countries were able to react based on a clear strategy that aimed at achieving short term goals of preventing severe illness and deaths caused by the virus, intermediate goals of mitigating the socio-economic burdens due to the pandemic, and long-term goals of working proactively to secure the required doses of vaccines and exploring the long road of local manufacturing capacities in the continent. This pandemic could be a turning point in the preparedness and resilience of the African health systems. A major step towards coordinating the efforts and sharing experiences made it easier to implement the health security strategies across the continent to achieve health, well-being, and prosperity to African citizens.

## Data Availability

Publicly available datasets were analyzed in this study. This data can be found here: [https://ourworldindata.org/covid-vaccinations?country=OWID_WRL/, https://africacdc.org/covid-19-vaccination/, accessed on 10 September 2021].

## References

[1] This Is Not a Drill: 5 Reasons Why the Experts are Worried about the Next Pandemic|Pursuit by The University of Melbourne. https://pursuit.unimelb.edu.au/articles/this-is-not-a-drill-5-reasons-why-the-experts-are-worried-about-the-next-pandemic.

[2] Allen T., Murray K.A., Zambrana-Torrelio C., Morse S.S., Rondinini C., di Marco M., Breit N., Olival K.J., Daszak P. (2017). Global Hotspots and Correlates of Emerging Zoonotic Diseases. Nat. Commun..

[3] Bouzid M., Colón-González F.J., Lung T., Lake I.R., Hunter P.R. (2014). Climate Change and the Emergence of Vector-Borne Diseases in Europe: Case Study of Dengue Fever. BMC Public Health.

[4] Fenollar F., Mediannikov O. (2018). Emerging Infectious Diseases in Africa in the 21st Century. New Microbes New Infect..

[5] 2014 Ebola Virus Outbreak: Facts, Symptoms, and How to Help|World Vision. https://www.worldvision.org/health-news-stories/2014-ebola-virus-outbreak-facts.

[6] Ebola: The Failures of the International Outbreak Response|MSF. https://www.msf.org/ebola-failures-international-outbreak-response.

[7] Piot P., Soka M.J., Spencer J. (2019). Emergent Threats: Lessons Learnt from Ebola. Int. Health.

[8] The Race for an Ebola Vaccine|The New Yorker. https://www.newyorker.com/business/currency/race-ebola-vaccine.

[9] Kieny M.P. (2018). Lessons Learned from Ebola Vaccine R&D during a Public Health Emergency. Hum. Vaccines Immunother..

[10] Ferorelli D., Spagnolo L., Marrone M., Corradi S., Silvestre M., Misceo F., Bianchi F.P., Stefanizzi P., Solarino B., Dell’erba A. (2021). Off-Label Use of COVID-19 Vaccines from Ethical Issues to Medico-Legal Aspects: An Italian Perspective. Vaccines.

[11] TASS WHO Reports over 120 Million Cases of COVID-19 Cases Worldwide. https://tass.com/world/1267279.

[12] Noh J., Danuser G. (2021). Estimation of the Fraction of COVID-19 Infected People in U.S. States and Countries Worldwide. PLoS ONE.

[13] Study: COVID Cases Have Been ‘Severely Undercounted’. https://www.webmd.com/lung/news/20210209/study-says-covid-cases-have-been-severely-undercounted.

[14] The Best Global Responses to COVID-19 Pandemic, 1 Year Later|Time. https://time.com/5851633/best-global-responses-covid-19/.

[15] Zhu H., Wei L., Niu P. (2020). The Novel Coronavirus Outbreak in Wuhan, China. Glob. Health Res. Policy.

[16] Emergency Use Authorization for Vaccines Explained|FDA. https://www.fda.gov/vaccines-blood-biologics/vaccines/emergency-use-authorization-vaccines-explained.

[17] COVID-19: Egypt Confirms First Coronavirus Case in Africa|Africanews. https://www.africanews.com/2020/02/14/covid-19-egypt-confirms-first-coronavirus-case-in-africa/.

[18] Massinga Loembé M., Tshangela A., Salyer S.J., Varma J.K., Ouma A.E.O., Nkengasong J.N. (2020). COVID-19 in Africa: The Spread and Response. Nat. Med..

[19] Africa Facts, Capital Cities, Currency, Flag, Language, Landforms, Land Statistics, Largest Cities, Population, Symbols. https://www.worldatlas.com/webimage/countrys/affacts.htm.

[20] (2021). Population of Africa. https://www.worldometers.info/world-population/africa-population/.

[21] Charts of the Week: Africa’s Changing Demographics. https://www.brookings.edu/blog/brookings-now/2019/01/18/charts-of-the-week-africas-changing-demographics/.

[22] Nations Online Project Official and Spoken languages of African Countries. https://www.nationsonline.org/oneworld/african_languages.htm.

[23] List of conflicts in Africa. Military Wiki—Fandom. https://military.wikia.org/wiki/List_of_conflicts_in_Africa.

[24] Addis A.K., Zhu Z. (2018). The Political Situation, Trends and Geopolitical Implications of Sub-Saharan and North African Countries: Comparative Study. Open J. Polit. Sci..

[25] Covid-19 Takes Its Toll on African Economy. Africanews. https://www.africanews.com/2020/12/30/covid-19-takes-its-toll-on-african-economy/.

[26] Zeufack A.G., Calderon C., Kambou G., Djiofack C.Z., Kubota M., Korman V., Cantu Canales C. (2020). Africa’s Pulse, No. 21, Spring 2020: An Analysis of Issues Shaping Africa’s Economic Future.

[27] Six Charts Show the Challenges Faced by Sub-Saharan Africa. https://www.imf.org/en/News/Articles/2021/04/12/na041521-six-charts-show-the-challenges-faced-by-sub-saharan-africa.

[28] Amid Recession, Sub-Saharan Africa Poised for Recovery. https://www.worldbank.org/en/news/press-release/2021/03/31/amid-recession-sub-saharan-africa-poised-for-recovery.

[29] Craig J., Hauck S. Estimates of Critical Care Capacity in 54 African Countries. Center for Disease Dynamics, Economics & Policy (CDDEP). https://cddep.org/publications/critical-care-capacity-africa/.

[30] Sustainable Development Goals. United Nations Development Programme. https://www.undp.org/sustainable-development-goals#good-health.

[31] Learn More About COVID-19 Vaccines From the FDA. https://www.fda.gov/consumers/consumer-updates/learn-more-about-covid-19-vaccines-fda.

[32] Draft Landscape and Tracker of COVID-19 Candidate Vaccines. https://www.who.int/publications/m/item/draft-landscape-of-covid-19-candidate-vaccines.

[33] Would Exempting COVID-19 Vaccines from Intellectual Property Rights Improve Global Access and Equity?. https://www.cgdev.org/debate/would-exempting-covid-19-vaccines-intellectual-property-rights-improve-global-access.

[34] Africa CDC COVID-19 Vaccination. https://africacdc.org/covid-19-vaccination/.

[35] Coronavirus (COVID-19) Vaccinations. https://ourworldindata.org/covid-vaccinations?country=OWID_WRL.

[36] Africa CDC Outbreak Brief 76: Coronavirus Disease 2019 (COVID-19) Pandemic. https://africacdc.org/download/outbreak-brief-76-coronavirus-disease-2019-covid-19-pandemic/.

[37] Haider N., Osman A.Y., Gadzekpo A., Akipede G.O., Asogun D., Ansumana R., Lessells R.J., Khan P., Hamid M.M.A., Yeboah-Manu D. (2020). Lockdown Measures in Response to COVID-19 in Nine Sub-Saharan African Countries. BMJ Glob. Health.

[38] Africa CDC Africa Joint Continental Strategy for COVID-19 Outbreak. https://africacdc.org/download/africa-joint-continental-strategy-for-covid-19-outbreak/.

[39] Africa CDC Partnership to Accelerate COVID-19 Testing (PACT) in Africa. https://africacdc.org/download/partnership-to-accelerate-covid-19-testing-pact-in-africa/.

[40] Africa Medical Supplies Platform ABOUT US. https://amsp.africa/about-us/.

[41] African Union AMSP Ppens COVID-19 Vaccines Pre-Orders for 55 African Union Member States. https://au.int/en/pressreleases/20210114/amsp-opens-covid-19-vaccines-pre-orders-55-african-union-member-states.

[42] Africa CDC Virtual Conference: Expanding Africa’s Vaccine Manufacturing. https://africacdc.org/event/virtual-conference-expanding-africas-vaccine-manufacturing/.

[43] Africa CDC African Union and Africa CDC Launches Partnerships for African Vaccine Manufacturing (PAVM), Framework to Achieve It and Signs 2 MoUs. https://africacdc.org/news-item/african-union-and-africa-cdc-launches-partnerships-for-african-vaccine-manufacturing-pavm-framework-to-achieve-it-and-signs-2-mous/.

[44] African Union African Union Receives Donation of Medical Supplies from Government of Canada to Enhance COVID-19 Response. https://au.int/en/pressreleases/20200730/au-receives-donation-medical-supplies-gov-canada-enhance-covid-19-response.

[45] Africa CDC Africa CDC Receives Third Donation of Medical Supplies from Jack Ma Foundation, Co-Hosts Global MediXChange Webinar on COVID-19. https://africacdc.org/news-item/africa-cdc-receives-third-donation-of-medical-supplies-from-jack-ma-foundation-co-hosts-global-medixchange-webinar-on-covid-19/.

[46] The Lancet (2020). An African Plan to Control COVID-19 Is Urgently Needed. Lancet.

[47] Africa CDC COVID-19 Vaccine Development and Access Virtual Conference. https://africacdc.org/news-item/covid-19-vaccine-development-and-access-virtual-conference/.

[48] Africa CDC COVID-19 Vaccine Development and Access Strategy. https://africacdc.org/download/covid-19-vaccine-development-and-access-strategy/.

[49] Africa CDC Africa’s Leadership Role in Covid-19 Vaccine Development and Access. https://africacdc.org/event/africas-leadership-role-in-covid-19-vaccine-development-and-access/.

[50] Africa CDC Framework for Fair, Equitable and Timely Allocation of COVID-19 Vaccines in Africa. https://africacdc.org/download/framework-for-fair-equitable-and-timely-allocation-of-covid-19-vaccines-in-africa/.

[51] Africa CDC AMSP Opens COVID-19 Vaccines Pre-Orders for 55 African Union Member States. https://africacdc.org/news-item/amsp-opens-covid-19-vaccines-pre-orders-for-55-african-union-member-states/.

[52] Africa CDC Statement on Donation and Distribution of Oxford-AstraZeneca COVID-19 Vaccine through AVATT. https://africacdc.org/news-item/statement-on-donation-and-distribution-of-oxford-astrazeneca-covid-19-vaccine-through-avatt/.

[53] Africa CDC Statement to African Union Member States on the Deployment of the AstraZeneca COVID-19 Vaccine to the Continent and Concerns about Adverse Event Reports Coming from Europe. https://africacdc.org/news-item/statement-to-african-union-member-states-on-the-deployment-of-the-astrazeneca-covid-19-vaccine-to-the-continent-and-concerns-about-adverse-event-reports-coming-from-europe/.

[54] African Export-Import Bank Africa Signs Historic Agreement with Johnson & Johnson for 400 Million Doses of COVID-19 Vaccines. https://www.afreximbank.com/africa-signs-historic-agreement-with-johnson-johnson-for-400-million-doses-of-covid-19-vaccines/.

[55] Africa Looks to Kickstart COVID Vaccine Production. https://www.dw.com/en/africa-covid-vaccine-production-moderna-biontech/a-59639332.

[56] COVAX Facility. https://www.gavi.org/covax-facility.

[57] World Health Organization (2021). COVID-19 National Deployment and Vaccination Plan Submission and Review Process.

[58] African Countries Have an Advantage in Rolling Out Covid-19 Vaccines. https://www.gavi.org/vaccineswork/african-countries-have-advantage-rolling-out-covid-19-vaccines.

[59] Herzog L.M., Norheim O.F., Emanuel E.J., McCoy M.S. (2021). Covax Must Go beyond Proportional Allocation of Covid Vaccines to Ensure Fair and Equitable Access. BMJ.

[60] Fair Allocation Mechanism for COVID-19 Vaccines through the COVAX Facility. https://www.who.int/publications/m/item/fair-allocation-mechanism-for-covid-19-vaccines-through-the-covax-facility.

[61] The Access to COVID-19 Tools (ACT) Accelerator. https://www.who.int/initiatives/act-accelerator.

[62] What Is the ACT Accelerator. https://www.who.int/initiatives/act-accelerator/about.

[63] ACT—A Prioritized Strategy and Budget for 2021. https://www.who.int/publications/m/item/act-a-prioritized-strategy-and-budget-for-2021.

[64] What Is the Access to COVID-19 Tools (ACT) Accelerator, How Is It Structured and How Does It Work?. https://www.who.int/publications/m/item/what-is-the-access-to-covid-19-tools-(act)-accelerator-how-is-it-structured-and-how-does-it-work.

[65] Gavi COVAX AMC. https://www.gavi.org/gavi-covax-amc.

[66] The Gavi COVAX AMC Explained. https://www.gavi.org/vaccineswork/gavi-covax-amc-explained.

[67] First COVID-19 COVAX Vaccine Doses Administered in Africa. https://www.unicef.org/press-releases/first-covid-19-covax-vaccine-doses-administered-africa.

[68] Ghana Is First Nation to Get Free Covid-19 Vaccines Under Covax Plan. https://www.wsj.com/articles/first-free-covid-vaccines-from-who-backed-covax-arrive-in-ghana-11614155319.

[69] Ghana Faces Hurdles to Achieve Targets Set for COVID-19 Vaccine Rollout. https://theconversation.com/ghana-faces-hurdles-to-achieve-targets-set-for-covid-19-vaccine-rollout-155075.

[70] COVID-19 Vaccines. https://www.afro.who.int/health-topics/coronavirus-covid-19/vaccines.

[71] Coronavirus: Africa’s Vaccination Rollout off to Slow Start. https://www.dw.com/en/coronavirus-africas-vaccination-rollout-off-to-slow-start/a-57242006.

[72] India’s Halt to Vaccine Exports “Very Problematic” for Africa. https://www.reuters.com/business/healthcare-pharmaceuticals/indias-halt-vaccine-exports-very-problematic-africa-2021-05-18/.

[73] Muna W. It’s Crucial for Africa to Manufacture More of Its Own Vaccines. https://www.chinadaily.com.cn/a/202107/30/WS61035142a310efa1bd6656de.html.

[74] Less Than 2% of World’s COVID-19 Vaccines Administered in Africa. https://www.afro.who.int/news/less-2-worlds-covid-19-vaccines-administered-africa.

[75] Africa: COVID-19 Vaccination Rate by Country 2021. https://www.statista.com/statistics/1221298/covid-19-vaccination-rate-in-african-countries/.

[76] US COVID Cases Drop Another 30% as Africa Surge Continues. https://www.cidrap.umn.edu/news-perspective/2021/06/us-covid-cases-drop-another-30-africa-surge-continues.

[77] Africa Sees 44% Spike in New Covid Infections, 20% Increase in Deaths. https://www.cnbc.com/2021/06/16/africa-sees-44percent-spike-in-new-covid-infections-20percent-increase-in-deaths-.html.

[78] Mathieu E., Ritchie H., Ortiz-Ospina E., Roser M., Hasell J., Appel C., Giattino C., Rodés-Guirao L. (2021). A Global Database of COVID-19 Vaccinations. Nat. Hum. Behav..

[79] Callaway E. (2021). Coronavirus Variants Get Greek Names—But Will Scientists Use Them?. Nature.

[80] Lessons for Africa from India’s Deadly COVID Surge [EN/AR/PT]. https://reliefweb.int/report/world/lessons-africa-india-s-deadly-covid-surge-enarpt.

